# The potential impact of preventive therapy against tuberculosis in the WHO South-East Asian Region: a modelling approach

**DOI:** 10.1186/s12916-020-01651-5

**Published:** 2020-07-20

**Authors:** Sandip Mandal, Vineet Bhatia, Mukta Sharma, Partha Pratim Mandal, Nimalan Arinaminpathy

**Affiliations:** 1 Indian Council of Medical Research, New Delhi, India; 2 South-East Asian Regional Office, World Health Organization, New Delhi, India; 3 MRC Centre for Global Infectious Disease Analysis, School of Public Health, Imperial College London, London, UK

**Keywords:** Preventive therapy, Epidemiology, Modelling, South East Asia, Tuberculosis

## Abstract

**Background:**

The prevention of tuberculosis (TB) is key for accelerating current, slow declines in TB burden. The 2018 World Health Organization (WHO) guidelines on eligibility for preventive therapy to treat latent TB infection (LTBI) include people living with human immunodeficiency virus (PLHIV), household contacts of TB patients including children, and those with clinical conditions including silicosis, dialysis, transplantation, etc. and other country-specific groups. We aimed to estimate the potential impact of full implementation of these guidelines in the WHO South-East Asian (SEA) Region, which bears the largest burden of TB and LTBI amongst the WHO regions.

**Methods:**

We developed mathematical models of TB transmission dynamics, calibrated individually to each of the 11 countries in the region. We modelled preventive therapy in the absence of other TB interventions. As an alternative comparator, reflecting ongoing developments in TB control in the region, we also simulated improvements in the treatment cascade for active TB, including private sector engagement and intensified case-finding. Relative to both scenarios, for each country in the region, we projected TB cases and deaths averted between 2020 and 2030, by full uptake of preventive therapy, defined as comprehensive coverage amongst eligible populations as per WHO guidelines, and assuming outcomes consistent with clinical trials. We also performed sensitivity analysis to illustrate impact under less-than-optimal conditions.

**Results:**

At the regional level, full uptake of preventive therapy amongst identified risk groups would reduce annual incidence rates in 2030 by 8.30% (95% CrI 6.48–10.83) relative to 2015, in the absence of any additional interventions. If implemented against a backdrop of improved TB treatment cascades, preventive therapy would achieve an incremental 6.93 percentage points (95% CrI 5.81–8.51) of reduction in annual incidence rates, compared to 2015. At the regional level, the numbers of individuals with latent TB infection that need to be treated to avert 1 TB case is 64 (95% CrI 55–74). Sensitivity analysis illustrates that results for impact are roughly proportional to ‘effective coverage’ (the product of actual coverage and effectiveness of the regimen).

**Conclusions:**

Full implementation of WHO guidelines is important for ending TB in the SEA Region. Although future strategies will need to be expanded to the population level, to achieve large declines in TB incidence, the uptake of current tools can offer a valuable step in this direction.

## Background

Despite large-scale efforts to improve TB services over the last two decades, global TB burden today is decreasing at a rate of only 1–2% per year. There remain critical challenges in the TB treatment cascade, for example, limited outreach of service, and missed opportunities for diagnosis in many high-burden settings [1, 2]. However, it is widely recognised that prevention, as well as optimising TB diagnosis and treatment, will be critical in any strategy aimed towards ending TB [3–5].

Despite ongoing vaccine development [6, 7] and increasing attention on the social determinants of TB [8, 9], preventive therapy arguably remains the primary option immediately available for TB prevention. In the absence of a widely deployable test to identify who would benefit most from preventive therapy, World Health Organization (WHO) guidelines identify high-risk groups for eligibility: for example, those with human immunodeficiency virus (HIV) coinfection [10] and, in the most recently updated guidelines, all household contacts of diagnosed TB cases and those with clinical conditions including silicosis, those on anti-TNF treatment, and other country-specific groups [11]. However, global uptake has been slow, partly owing to challenges in initiating and administering preventive therapy regimens, lasting several months, amongst otherwise healthy individuals [12, 13]. In the WHO South-East Asian Region, only 15% of PLHIV and 26% of eligible children < 5 years of age were reported to have received preventive treatment in 2018 [14].

Nonetheless, options for preventive therapy have seen important developments in recent years. Isoniazid preventive therapy (IPT) involves 6 months daily treatment with isoniazid, and could lower the risk of active TB by 60%, amongst those with latent TB infection [15]. More recent developments include 3HR (isoniazid and rifampicin daily for 3 months), 3HP (isoniazid and rifapentine once weekly for 3 months) and 1HP (isoniazid and rifapentine once weekly for 1 month), all regimen options achieving non-inferior effectiveness to IPT, with shortened and simplified treatment [16–18]. These and future regimens may offer new opportunities to accelerate uptake of TB prevention, in line with WHO recommendations.

In this context, strategic planning could benefit from estimation of the potential epidemiological impact of preventive therapy. In the present work, we aimed to address this need, using dynamical mathematical models. We concentrated on the WHO South-East Asian (SEA) Region [19], estimated to account for > 30% of global prevalence of latent TB infection and 44% of global TB incidence, the highest of any of the WHO regions [4, 14]. We developed mathematical models of TB transmission, calibrated to the TB epidemic in each of the 11 countries in the region. Using this framework, we estimated the potential reductions in TB incidence and mortality that could be achieved by 2030, with implementation of WHO guidelines in each of the 11 countries in the region.

## Methods

### Model structure

The WHO SEA Region consists of 11 countries: Bangladesh, Bhutan, DPR Korea, India, Indonesia, Maldives, Myanmar, Nepal, Sri Lanka, Thailand and Timor-Leste. We built on a deterministic, compartmental framework developed in earlier work, in support of strategic planning in the region [20]. Here we outline the model structure and approach, with further technical details given in the supplementary document (See Additional file 1 [1, 11, 14, 21–32]).

**Fig. 1:**
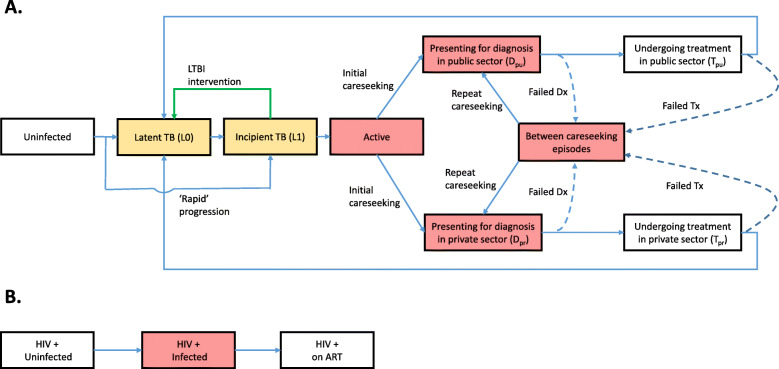
Illustration of the model structure. Panel **a** shows the compartmental model framework representing TB natural history and the uptake of TB services. Abbreviations: ‘Dx’ denotes diagnosis; ‘Tx’ denotes ‘treatment’. The structure shown here is further stratified by HIV status, according to the categories shown in panel **b** and by rifampicin resistance (not shown here for clarity). Further technical details are provided in the supporting information

The model structure is illustrated schematically in Fig. 1. A characteristic feature of many countries in the SEA region is the presence of a large private healthcare sector, particularly in Bangladesh, India, Indonesia, Myanmar, Nepal and Thailand. In these countries, the private sector plays a major role in TB care, often not reporting TB cases to public health authorities [33–37]. There is evidence to indicate that TB care in the private sector does not uniformly meet national standards [36, 38], leading to delays in diagnosis and suboptimal treatment outcomes. Accordingly, in the model, we distinguished between public and private sectors, allowing for a lower standard of diagnosis and less favourable treatment outcomes in the latter than the former (see Tables S3 and S4 for parameters in Additional file 1 [1, 14, 15, 17, 26, 27, 31, 32, 39–49]).

### Calibration and uncertainty

We calibrated the model independently for each of the 11 SEA regional countries, to WHO estimates of TB burden from 2014 to 2018, the estimated proportion of TB cases having HIV coinfection in 2018, and WHO data for public sector notifications in 2018 (see Table S2 in Additional file 1 [14]). We combined this data with uncertainty ranges for model parameters (see Table S3 for parameter ranges in Additional file 1 [1, 14, 15, 17, 26, 27, 31, 32, 39–49]), ultimately using Bayesian Markov-Chain Monte-Carlo (MCMC) [21] methods, a technique widely used in modelling studies [22–24] to sample from the posterior distribution of model parameters. As described below, we then used these samples to create model projections for the future impact of preventive therapy. This method allows a systematic approach for propagating uncertainty from model inputs to model outputs. For each country, we conducted the MCMC and recorded 10,000 iterations following initial ‘burn-in’. For any given model projection, we estimated 95% credible intervals as the 2.5th and 97.5th percentiles arising from these samples.

### Modelling preventive therapy

WHO guidelines for preventive therapy have adapted over time, incorporating steadily expanding eligibility criteria. The most recent guidelines identify as priorities for preventive therapy: those living with HIV, HIV-negative contacts of TB patients (prioritising < 5 years old but also including adult and adolescent contacts), in whom active TB disease has been ruled out; and those with other clinical risk factors, including silicosis, transplant patients, and those receiving dialysis [11]. In the present analysis, we defined ‘full uptake’ as a scenario where (i) all eligible individuals receive preventive therapy, and (ii) where the outcomes of preventive therapy are consistent with those observed in clinical trials, assuming that both of these conditions are achieved over a 3-year scale-up period. We also performed sensitivity analysis to examine how the modelled impact would vary under less optimal conditions for coverage and effectiveness of preventive therapy.

HIV coinfection is a prominent risk factor for developing TB, elevating a 10% lifetime risk of developing TB (amongst HIV-negative individuals) to a 10% annual risk [10]. Preventive therapy has been shown to mitigate this risk by up to 60% [25]. In the SEA region, HIV accounted for an estimated 3.2% of TB incidence in 2018, on the country level ranging from 0% in Maldives to 8.5% in Thailand. Accordingly, we modelled the impact of preventive therapy amongst people living with HIV by a reduced rate of progression to active disease, reflecting both the coverage and efficacy of preventive therapy (see Additional file 1 [11, 26, 27, 29–32]).

HIV-negative household contacts are an important risk group because (i) the prevalence of latent TB infection in households of TB patients can be twice as high as in the general community, and (ii) those with latent infection are likely to have been recently infected with TB, the interval in which they are at highest risk of developing active disease [11]. As a result of both factors, the incidence rate of TB diseases amongst a cohort of household contacts with LTBI is substantially greater than in the general population. To quantify this relative risk, we drew from a recent case-finding study in Vietnam [26] that conducted longitudinal follow-up amongst household contacts of TB patients, finding an incidence of 894 per 100,000 contacts in the first year of follow-up, 7 times greater than WHO estimates of country-level incidence in Vietnam. This rate ratio is consistent with findings from ongoing household cohort studies in India [27].

It is not feasible to model households explicitly within a compartmental modelling framework. Rather than aiming to capture this type of population structure, we asked the question: ‘What are the population-level implications of reducing TB incidence amongst a defined cohort (of household contacts) in the population?’. We first estimated the *direct* effect on incidence: that is, the reduction in incidence that would arise from a given coverage of preventive therapy amongst household contacts, in the absence of secondary transmission effects. Subsequently, and to capture indirect (transmission-mediated) effects, we incorporated this direct effect into the transmission model, in order to simulate the full incidence reductions at the population level. Approaches for estimating direct and indirect effects have been described previously [28] and, in brief, involve controlling for the force-of-infection under different scenarios. Further technical details are given in the supplementary document (see Additional file 1). We modelled other clinical risk factors (e.g., silicosis and receiving dialysis) as an aggregate group and using a similar approach as that described above for household contacts.

We simulated all scenarios from 2018 to 2030, assuming preventive therapy coverage to be scaled up in a linear way over 3 years from 2020 to 2023. We captured current levels of preventive therapy coverage in each country, amongst both household contacts and PLHIV (at the regional level, current coverages of 26% and 15%, respectively); we assumed that the intervention would gradually replace existing IPT with new, rifamycin-based shorter regimens [17] as per eligibility criteria when scaling up to cover all household contacts of notified TB cases, as well as to cover all PLHIV.

To model the impact of preventive therapy, it is also important to model what may have occurred in future, in the absence of this intervention (the ‘comparator’ scenario, against which the incremental impact of preventive therapy is assessed). We adopted two comparators: (i) a ‘status quo’ comparator, with current TB services continuing without change indefinitely, and (ii) an ‘improved TB cascade’ comparator to capture ongoing efforts across the region to improve TB control, independent of preventive therapy. The latter includes comprehensive scale-up of services including engagement of the private/outside the national TB programmes (non-NTP sector), along with improvement in TB diagnostics and treatment outcomes, and additional efforts to accelerate the diagnosis of active TB, including measures such as intensified case-finding and generation of demand for TB services [50, 51]. Modelled in previous work [20], these scenarios are described in more detail in the supporting information. In the context of each of these comparators, we measured the impact of preventive therapy by projecting annual incidence and mortality rates in 2030, and comparing with rates in 2015, in line with the WHO End TB strategy [52].

Preventive therapy is complicated by drug-resistant infection, as these infections may be resistant to standard preventive therapy regimens, and it is currently not possible to test the drug sensitivity status of LTBI. As a conservative approach, we modelled only the use of a 3HP-like regimen amongst household contacts and amongst PLHIV, assuming that all those with drug-resistant LTBI would either be ruled out for preventive therapy or would not respond to it. This assumption tends to underestimate overall impact, as in practice, contacts of drug-resistant TB patients may be offered fluoroquinolone-based preventive regimens [11].

Finally, to identify the most influential model parameters, we selected a focal model output: the percent reduction in incidence rates as a result of full uptake of preventive therapy, in 2030 relative to 2015. We computed the partial rank correlation of this outcome against all model parameters (e.g. as described in ref. [53]), aiming to identify the most influential parameter as that showing the greatest correlation.

## Results

Figures S1 and S2 in the supplementary document (see Additional file 1) show results of model calibrations for each of the 11 countries in the region, and Tables S3, S4 shows the Bayesian posterior estimates for model parameters.

**Fig. 2:**
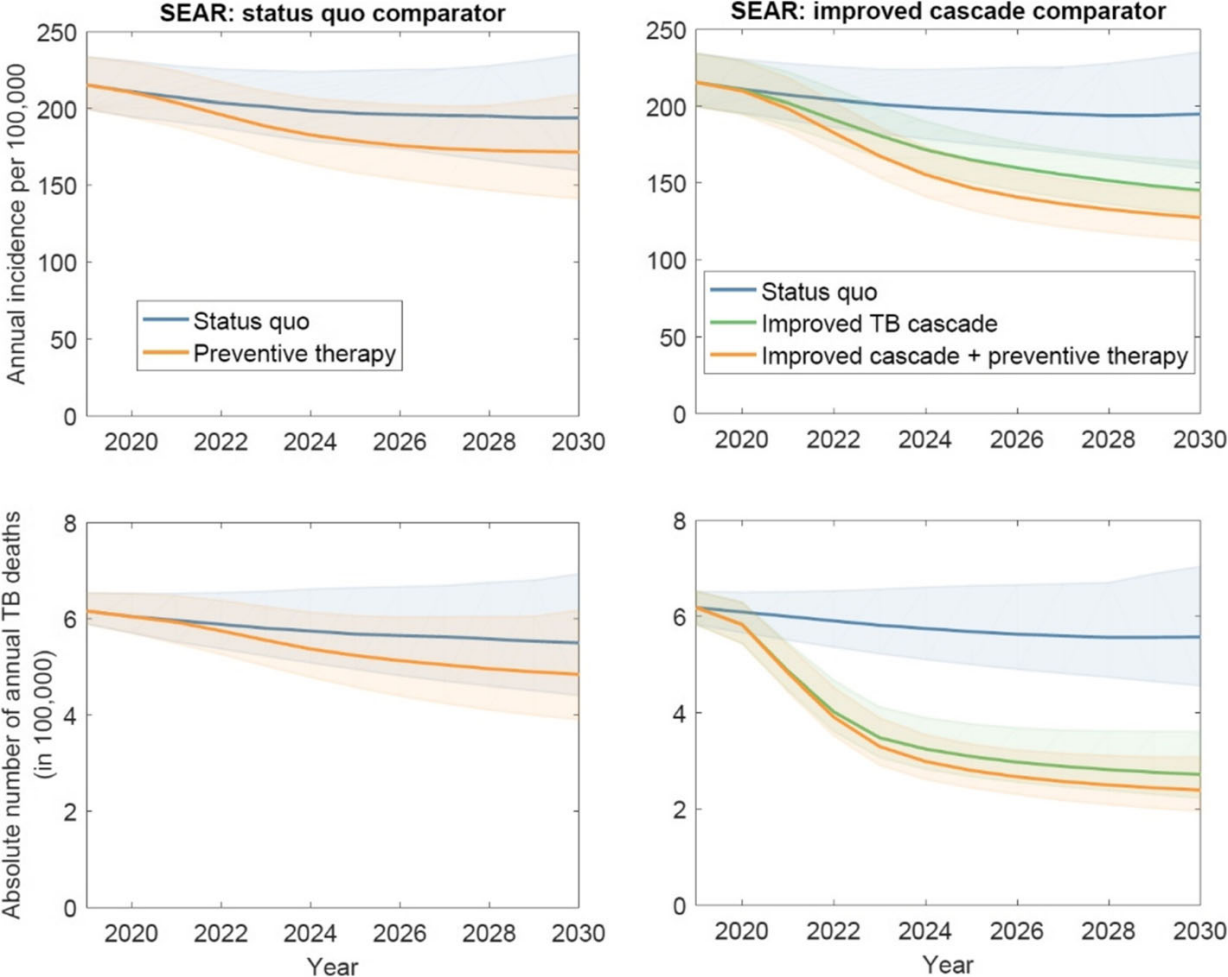
Model projections for incidence impact in SEAR, under adoption of WHO guidelines for management of LTBI in the region. See Table 1 for impact by country, as well as in terms of incidence and mortality. Shaded intervals show 95% Bayesian credible intervals. As described in the main text, the ‘status quo comparator represents current TB services continuing indefinitely without change, while the ‘Improved cascade’ comparator incorporates background improvements in TB care, including comprehensive engagement with the private healthcare sector, and intensified case-finding, throughout the region

Figure 2 shows model projections for incidence and mortality impact in the region, as an aggregate of projections over all 11 countries in the region, under the two comparator scenarios described above. In the absence of other additional TB interventions (‘status quo’ comparator), full adoption of WHO guidelines would result in a 8.30% (95% CrI 6.48–10.83) reduction of the annual incidence rate in 2030 relative to 2015, and a reduction of TB deaths in 2030 by 6.75% (95% CrI 5.19–8.54) relative to 2015. In the context of background interventions to improve TB services (the ‘improved TB cascade’ comparator), the incremental impact from preventive therapy would be to reduce incidence rates in 2030 by a further 6.93% (95% 5.81–8.51) and TB deaths in 2030 by a further 3.52% (95% 2.72–5.08) (both relative to 2015). Table 1 shows these results by country, while Tables S5 and S6 (Additional file 1) show impact in terms of *cumulative* cases and TB deaths averted. While these results focus on the incremental impact attributable to preventive therapy alone, Table S7 in the supporting information also shows the combined impact of improving the TB cascade and implementing preventive therapy.

**Table 1:** Impact on TB incidence and mortality, by country and for the whole region, by 2030 relative to 2015. Estimates show the incremental impact attributable to preventive therapy alone, in the context of each comparator. Thus estimates under the ‘status quo’ comparator reflect the difference between the blue and orange curves in the left-hand panel of Fig. 2 and estimates under the ‘improved TB cascade’ comparator reflect the difference between the green and yellow curves in the right-hand panel of Fig. 2

	Incremental impact of preventive therapy relative to ‘Status quo comparator’		Incremental impact of preventive therapy relative to ‘improved TB cascade’ comparator	
	% reduction in annual incidence rate (2030 relative to 2015)	% reduction in annual TB deaths (2030 relative to 2015)	% reduction in annual incidence rate (2030 relative to 2015)	% reduction in annual TB deaths (2030 relative to 2015)
Bangladesh	4.84 [4.33–8.14]	3.70 [1.62–7.79]	4.45 [3.79–5.60]	1.70 [0.57–4.21]
Bhutan	3.23 [2.76–6.58]	3.02 [0.99–7.79]	5.25 [2.66–4.25]	1.67 [0.43–4.63]
DPR Korea	15.90 [4.94–30.42]	15.60 [3.87–40.46]	8.79[−2.01–14.89]	4.80[− 1.08–14.94]
India	6.94 [5.04–10.64]	6.69 [4.56–9.47]	6.44 [5.08–8.33]	3.59 [2.48–6.07]
Indonesia	9.79 [7.23–14.13]	6.61 [4.83–8.50]	7.81 [6.52–10.05]	3.19 [2.41–4.60]
Maldives	3.45 [1.90–16.50]	0.14 [0.10–0.44]	1.97 [1.45–2.89]	0.07 [0.06–0.09]
Myanmar	10.59 [8.07–23.98]	8.25 [4.40–18.63]	12.41 [9.02–23.00]	6.02 [2.81–11.26]
Nepal	6.17 [4.56–13.68]	8.80 [3.34–23.87]	5.22 [4.12–7.78]	4.71 [1.17–13.02]
Sri Lanka	2.01 [1.58–3.00]	1.27 [0.64–3.39]	2.15 [1.68–2.83]	1.06 [0.38–2.29]
Thailand	13.94 [7.96–36.96]	7.25 [3.17–19.43]	9.06 [6.47–15.52]	3.03 [1.23–7.89]
Timor Leste	49.4 [28.74–84.77]	39.18 [16.70–92.83]	22.39 [12.56–40.23]	9.47 [2.75–30.10]
SEAR	8.30 [6.48–10.83]	6.75 [5.19–8.54]	6.93 [5.81–8.51]	3.52 [2.72–5.08]

Table 2 additionally stratifies impact amongst those living with HIV and amongst household contacts of TB cases, taking the example of the improved TB cascade comparator. At the regional level, preventive therapy limited to people living with HIV would reduce annual incidence rates in 2030 by 1.81% (95% 1.30–2.38), relative to 2015. A larger impact arises from limiting preventive therapy to household contacts, yielding an incidence rate reduction of 6.93% (95% 5.81–8.51); this latter group accounts for the bulk of epidemiological impact in the SEA region.

**Table 2:** Incremental impact of preventive therapy stratified by coverage in PLHIV, and household contacts of reported TB cases. Shown in the example of the ‘improved TB cascade’ comparator, results illustrate the overall impact arising from both of these eligible populations

	Targeting PLHIV (%)	Targeting household contacts (%)	Total (%)
Reduction in annual incidence rate attributable to preventive therapy (2030 relative to 2015), under the improved cascade comparator			
Bangladesh	0.09 [0.05–0.14]	4.38 [3.71–4.49]	4.45 [3.79–5.60]
Bhutan	0.10 [0.03–0.39]	3.17 [2.57–3.95]	3.25 [2.66–4.25]
DPR Korea	0.09 [0.04–0.17]	8.69[−2.07–14.81]	8.79[−2.01–14.89]
India	1.79 [1.14–2.91]	4.53 [3.56–5.79]	6.44 [5.08–8.33]
Indonesia	1.53 [0.85–2.99]	6.35 [5.50–7.99]	7.81 [6.52–10.05]
Maldives	0.03 [0.025–0.049]	1.94 [1.43–2.86]	1.97 [1.45–2.90]
Myanmar	4.39 [2.70–7.69]	7.96 [6.04–15.50]	12.41 [9.02–23.00]
Nepal	0.62 [0.44–0.94]	4.62 [3.60–6.96]	5.22 [4.12–7.78]
Sri Lanka	0.20 [0.13–0.27]	1.95 [1.49–2.65]	2.15 [1.68–2.83]
Thailand	5.69 [3.84–9.23]	3.40 [2.50–5.62]	9.06 [6.47–15.52]
Timor Leste	1.06 [0.44–2.64]	21.29 [12.18–38.06]	22.39 [12.56–40.23]
SEAR	1.81 [1.30–2.38]	5.14 [4.38–6.14]	6.93 [5.81–8.51]
Reduction in annual TB mortality attributable to preventive therapy (2030 relative to 2015), under the improved cascade comparator			
Bangladesh	0.03 [0.01–0.10]	1.67 [0.56–4.12]	1.70 [0.57–4.21]
Bhutan	0.05 [0.008–0.28]	1.57 [0.43–4.49]	1.67 [0.43–4.63]
DPR Korea	0.05 [0.02–0.14]	4.74[−1.13–14.81]	4.80[− 1.08–14.94]
India	1.05 [0.58–1.88]	2.62 [1.59–4.29]	3.59 [2.48–6.07]
Indonesia	0.61 [0.35–1.15]	2.57 [1.88–3.60]	3.19 [2.40–4.60]
Maldives	0.001 [0.0007–0.0013]	0.07 [0.06–0.09]	0.07 [0.06–0.09]
Myanmar	1.98 [0.93–3.79]	3.97 [1.76–7.67]	6.02 [2.81–11.26]
Nepal	0.53 [0.15–1.54]	4.18 [1.02–11.47]	4.71 [1.17–13.02]
Sri Lanka	0.09 [0.04–0.25]	0.97 [0.34–2.13]	1.06 [0.38–2.29]
Thailand	1.92 [0.78–5.12]	1.15 [0.52–3.36]	3.03 [1.23–7.89]
Timor Leste	0.39 [0.10–1.33]	9.00 [2.66–29.17]	9.47 [2.75–30.10]
SEAR	0.94 [0.58–1.45]	2.60 [1.96–3.73]	3.52 [2.72–5.08]

**Fig. 3:**
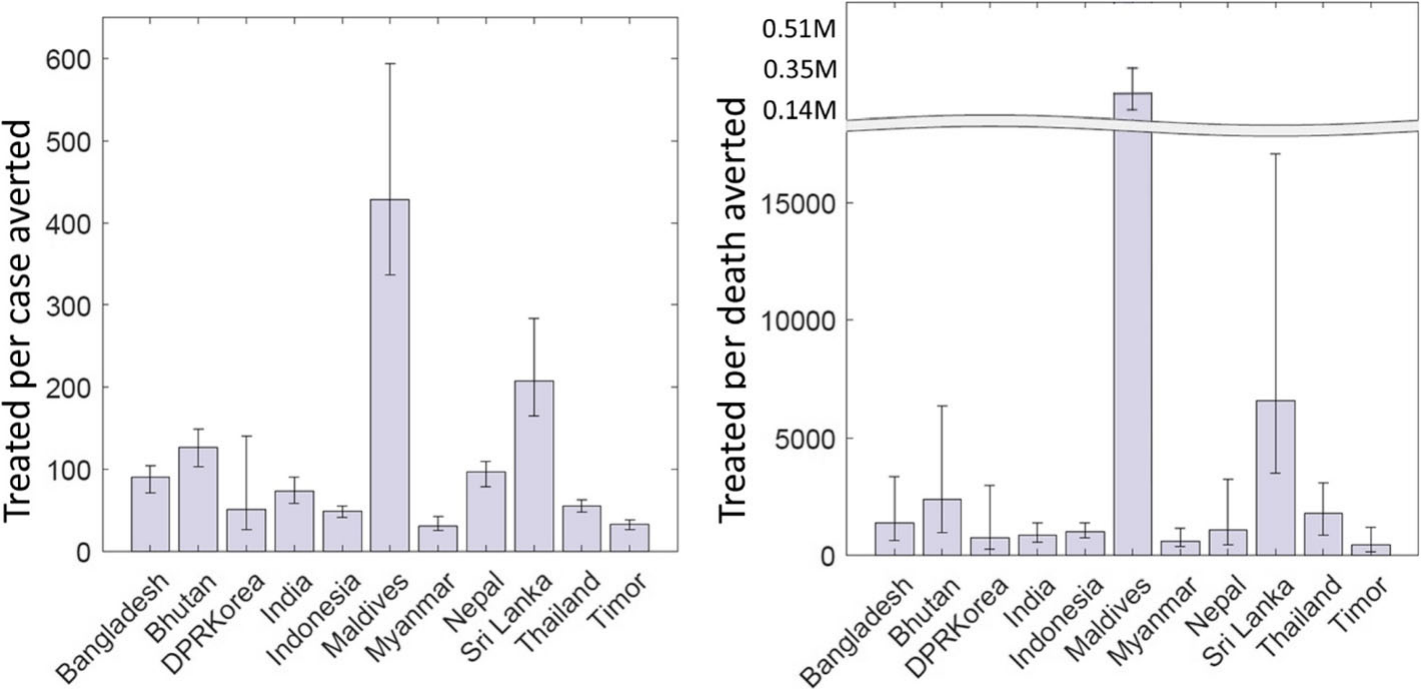
Numbers-needed-to-treat with preventive therapy, to prevent 1 TB case. Figure shows estimates stratified by the 11 countries in the region, as a simple proxy for the effort required to achieve the incidence declines shown in Fig. 2. Error bars show 95% Bayesian credible intervals. In the second panel, numbers-needed-to-treat are disproportionately high for the Maldives because of a low incidence (33 per 100 k population), as well as a low reported TB mortality rate (0.15 per 100 k population)

As a simple proxy for resource needs, we sought to estimate the numbers of individuals that would need to receive preventive therapy, in order to avert 1 TB case and to avert 1 TB death. Figure 3 shows model projections, illustrating these to vary widely by country setting. Numbers-needed-to-treat to avert 1 TB case are generally higher for low-burden countries (e.g. 208 (95% CrI 165–283) for Sri Lanka) than for higher-burden settings (e.g. 73 (95% CrI 59–91) for India), potentially because higher levels of transmission in higher-burden settings also amount to higher levels of indirect protection. Corresponding numbers to avert TB deaths are typically 10–20 times higher, for example with India needing 848 (95% CrI 552–1375) individuals to receive preventive therapy in order to avert 1 TB death.

Finally, Figs.S3, S4 in the supplementary document (see Additional file 1) shows results of sensitivity analysis. We first examined the model sensitivity to our assumption of ‘full uptake’ of preventive therapy, meaning that all eligible groups receive preventive therapy, with efficacy similar to those observed in clinical trials. Figure S3 relaxes these assumptions, showing that incidence impact is roughly proportional to ‘effective coverage’, defined as the product of the coverage and effectiveness of preventive therapy. Figure S4 shows additional sensitivity analysis, using partial rank correlation coefficients to identify the model inputs that are most influential for model outcomes. Results illustrate that the key uncertainty is the incidence rate amongst household contacts, relative to the general population (*k*_*HH*_). Notably less sensitivity is attached to the incidence rate amongst those with silicosis, dialysis patients, etc. (*k*_*RG*_), despite the wide uncertainty intervals adopted for this population: despite the clear value of preventive therapy amongst these patients, their relatively small size, as a proportion of the overall population, limits their potential contribution to overall declines in TB burden.

## Discussion

With preventive therapy forming an important part of the end TB strategy worldwide, it is important to assess the impact that could be achieved with currently available tools. Our results suggest that, amongst the 11 countries of the SEA Region, full adoption of WHO guidelines could have a meaningful impact in reducing TB burden between now and 2030. In particular, our results suggest that such measures could reduce TB incidence and deaths in the region by 8.30% (95% CrI 6.48–10.83) and 6.75% (95% CrI 5.19–8.54), respectively.

The relatively modest contribution of prevention amongst PLHIV to a reduction in incidence is because HIV coinfection does not play the strong role in driving TB transmission in SEAR as it does, for example, in South Africa: at the regional level in SEAR, an estimated 3.4% of incident TB cases are HIV coinfected [14]. Nonetheless, given the sheer mortality toll caused by TB amongst those living with HIV and the proven effectiveness of preventive therapy in this group, TB prevention amongst PLHIV justifiably continues to be a public health priority in the region. Moreover, there is substantial variation between countries within the region, with an estimated 9.8% of incident TB cases in Thailand being HIV-coinfected. In such settings, prevention amongst PLHIV plays a notably stronger role than on the regional level (Table 2). Broadly, the relative reductions in TB burden that are achievable by preventive therapy appear greater in those countries with higher TB incidence rates (Table 1). If the impact of preventive therapy is amplified by its indirect effects (i.e. those mediated by transmission), then it might be expected that this impact is greatest in settings where transmission is strongest.

It will be important for any future scale-up of preventive therapy to be accompanied by monitoring for drug resistance. In the context of isoniazid preventive therapy (IPT), despite concerns that widespread use could drive the emergence of drug resistance, the available evidence suggests otherwise [54, 55]. The risks of newer regimens driving drug resistance may be still smaller than the risk for IPT, given that they involve combinations of drugs, such as 3HP. Nonetheless, continued surveillance for drug resistance will remain essential.

Household contacts account for the bulk of projected impact of preventive therapy in the region, but more is required in order to meet the ambitious goals of the End TB strategy by 2030. As argued in previous work, there is a need for future preventive strategies to move beyond specific risk groups, to achieve TB prevention on a population level [20]. Such levels of coverage are infeasible with currently available tools, for example with the unsuitability of current preventive regimens for widescale deployment [13, 56]. Nonetheless, with the advent of new diagnostics and therapies [17, 18], the full implementation of current guidelines would form a valuable transitional step to the deployment of future preventive strategies. Intersectoral approaches, such as addressing comorbidities including undernutrition and diabetes, are also likely to play an important role in the population-level prevention of TB [8, 57, 58].

As with any modelling analysis, our work has entailed several simplifications. We have ignored age structure, as well as averaging over pulmonary and extrapulmonary forms of TB. We have also not addressed subnational variation within countries with, for example, different states within India having widely varying levels of TB burden [59]. As noted above, compartmental models are not readily suited for capturing household structure in the population: we have sought to address this challenge, while maintaining the overall tractability of the modelling approach, by abstracting from household structure. Future work could aim to test these assumptions by (i) building the evidence basis for TB incidence amongst household contacts in different settings, particularly though longitudinal designs such as in ref. [26]; (ii) seeking to measure the community benefits of preventive therapy; and (iii) comparing against alternative modelling approaches, including individual-based modelling. We have additionally not addressed the very real implementation challenges of preventive therapy [25], assuming scenarios where these challenges are sufficiently overcome to realise the full potential of preventive therapy. To some extent, these assumptions may be justified with the emergence of new, simplified preventive therapy regimens [17, 18], but this remains to be demonstrated.

While a full costing analysis is outside the scope of the current study, we have nonetheless estimated a simple proxy, the number of individuals with latent TB infection who would need to be treated with preventive therapy, in order to avert 1 TB case (Fig. 3). An important area for future analysis is to perform a more systematic costing analysis, including not only the costs for testing and treating LTBI, but also the cost savings that would arise from prevention of future TB episodes. Important developments, including the recently announced price reductions for the 3-month regimen [60], will impact any assessment of cost-effectiveness of preventive therapy. Future costing analysis will also be invaluable in informing investment strategies in the implementation of preventive therapy strategies, as well as in the development of new diagnostics and regimens.

## Conclusions

In conclusion, there is a need to expand current TB efforts beyond the TB treatment cascade, to a comprehensive strategy incorporating TB prevention as well. Although current strategies are necessarily limited to specific risk groups, in the WHO SEA Region, they could nonetheless achieve as much incidence and mortality impact as parallel measures to optimise the TB treatment cascade. Looking to the future, full implementation of these current strategies will form an important stepping stone to more wide-ranging preventive measures. Potentially facilitated by new technologies for targeted prevention, such measures will form a critical component of collective efforts to accelerate reductions in global TB incidence.

## Supplementary information

**Additional file 1 : Figure S1**. Calibration results to incidence and mortality. **Figure S2**. Calibration results to additional indicators. **Figure S3**. Sensitivity analysis to assumptions for coverage and effectiveness of preventive therapy. **Figure S4**. Sensitivity analysis to model parameters. **Table S1**. List of state variables used in the model. **Table S2**. Epidemiological indicators for model calibration. **Table S3**. List of regional-level parameters used in the model. **Table S4**. Summary of country-wise parameter estimates. **Table S5**. Impact on cumulative TB incidence by country, and for the whole region, from 2019-2030. **Table S6**. Impact on cumulative TB mortality by country, and for the whole region, from 2019-2030. **Table S7**. Total impact of improved TB cascade and preventive therapy.

## Data Availability

All data generated or analysed during this study are included in this published article and its supplementary information files.

## Abbreviations

H: Isoniazid
LTBI: Latent tuberculosis infection
MCMC: Markov-Chain Monte Carlo
P: Rifapentine
PLHIV: People living with HIV
R: Rifampicin
SEA: South-East Asia
TB: Tuberculosis
WHO: World Health Organization
