# Supplementary material for: The potential impact of preventive therapy against tuberculosis in the WHO South-East Asian Region: a modelling approach
Source: BMC Med. 2020 Jul 20;18:163. doi: 10.1186/s12916-020-01651-5 (PMC7369473; doi:10.1186/s12916-020-01651-5)

**The potential impact of preventive therapy against tuberculosis in the WHO South-East Asian Region: a modelling approach**

Sandip Mandal, Vineet Bhatia, Mukta Sharma, Partha Pratim Mandal, Nimalan Arinaminpathy

Supporting information

**1. Model specification**

The model is governed by the following equations (see table S1 for definitions of state variables, and table S3 for parameter definitions and sources). First, for the states prior to a TB patient’s first visit to a provider, we have:

$$\begin{matrix} \dot{U} & = & b-U\sum_{s} \lambda_{s}-\mu U \\ \dot{L_{hs}} & = & \left( 1-f_{h} \right)\lambda_{s}\left[ U+c_{i}\sum_{s} \left( L_{hs}+R_{hs} \right) \right]+rM_{hs}-\left( g_{h}+\mu\right)L_{hs} \\ \dot{M_{hs}} & = & f_{h}\lambda_{s}\left[ U+c_{i}\sum_{s} \left( L_{hs}+R_{hs} \right) \right]+g_{h}L_{hs}-\left( r+h+\mu\right)M_{hs} \\ \dot{I}_{hs} & = & hM_{hs}+\rho R_{hs}-\left( c+\sigma+\mu_{TB} \right)I_{hs} \end{matrix}$$

where subscript *h* denotes the HIV status (denoting ‘HIV +ve’ and ‘HIV -ve’ populations) and *s* denotes the infecting strain (denoting drug-susceptible and drug resistant TB). Next, we assume that a proportion *p_q_* of patient visits are to a provider of type *q* (denoting NTP and non-NTP providers). We have, for those awaiting diagnosis with provider type *q*:

$$\begin{matrix} \dot{D}_{qhs} & = & cp_{q}I_{hs}-\left( d+\sigma+\mu_{TB} \right)D_{qhs} \end{matrix}$$

We assume that a proportion $u_{q}$ of TB patients visiting provider type *q* successfully initiate TB treatment (the remainder constituting missed diagnosis as well as initial loss to follow up, covered below). For those initiating first-line treatment, it is convenient to specify equations separately by drug-susceptible (*s* = 0) and drug-resistant (*s* = 1) status. Thus we have, for drug-susceptible TB:

$$\dot{F}_{qh,0}=du_{q}D_{qh,0}-\left( \tau^{\left( FL \right)}+\delta_{q}+\alpha+\sigma+\mu\right)F_{qh,0}$$

where $\alpha$ represents the per-capita hazard of acquisition of multi-drug-resistance while on first-line TB treatment, only applicable to drug-sensitive TB. For drug-resistant TB, we have:

$$\dot{F}_{qh,1}=du_{q}{(1-v_{q})D}_{qh,1}+\alpha F_{qh,0}-\left( \tau^{\left( FL \right)}+\delta_{q}+{\sigma+\mu}_{TB} \right)F_{qh,1}$$

where $v_{q}$ is the proportion of TB patients presenting to a provider of type *q* who undergo drug sensitivity testing at the point of TB diagnosis.

For second-line treatment (only for DR-TB), we have:

$$\dot{S}_{qh,1}=du_{q}v_{q}D_{qh,1}+\tau^{\left( FL \right)}w_{q}F_{qh,1}-\left( \tau^{\left( SL \right)}+\mu\right)S_{qh,1}$$

where $w_{q}$ represents the proportion of DR-TB patients with provider type *q* who are switched to second-line treatment after failing first-line treatment.

Next, the compartment *B* captures those patients who have dropped out of the care cascade and remain infectious, whether by failed diagnosis, loss to follow up, subsequent default, or failed treatment. We have, for *B*:

$$\dot{B}_{hs}=\sum_{q} \left[ d\left( 1-u_{q} \right)D_{qhs}+\delta_{q}F_{qhs}+\left( 1-p_{q}^{\left( SL \right)} \right)\tau^{\left( SL \right)}S_{qhs} \right]-\left( \gamma+\sigma+\mu_{TB} \right)B_{hs}$$

For those who have recovered from disease, whether from treatment or cure, we have:

$$\dot{R}_{hs}=\sum_{q} \left[ \tau^{\left( FL \right)}F_{qhs}+\tau^{\left( SL \right)}p_{q}^{\left( SL \right)}S_{qhs}+\sigma D_{qhs} \right]+\sigma\left( I_{hs}+B_{hs} \right)-\left( \mu+\rho\right)R_{hs}$$

Finally, for the forces-of-infection $\lambda_{0},\lambda_{1}$ for DS- and DR-TB respectively, we have:

$\lambda_{0}=\beta\left[ \left( I_{0s}+B_{0s} \right)+\sum_{q,0} D_{q0s} \right]+\kappa\beta\left[ \left( I_{1s}+B_{1s} \right)+\sum_{q,1} D_{q1s} \right]$, (*)

and likewise for $\lambda_{1}$, but with $\beta_{MDR}$ in place of $\beta$.

**Table S1. List of state variables used in the model.**

| Symbol | Meaning |
| --- | --- |
| *Indicators* | |
| *q* | Indicator variable for provider type: *q* = 0, 1, 2 respectively for NTP providers, non-NTP providers and ‘engaged’ non-NTP providers |
| *h* | Indicator variable for *HIV* status: *h* = 0, 1 respectively for HIV-negative and *HIV-*positive |
| *s* | Indicator variable for drug sensitivity status: *s* = 0, 1 respectively for DS- and DR-TB |
| *State Variables* | |
| $\boldsymbol{U}$ | Proportion uninfected |
| $\boldsymbol{L}_{\boldsymbol{hs}}$ | Proportion having *latent infection* with HIV status *h* and having strain *s* |
| $\boldsymbol{M}_{\boldsymbol{hs}}$ | Proportion with latent infection but at *imminent risk* of developing TB (within next two years), with HIV status *h* and strain *s* |
| $\boldsymbol{I}_{\boldsymbol{hs}}$ | Proportion having *active disease* that have not yet presented for care, with HIV status *h* and strain *s* |
| $\boldsymbol{D}_{\boldsymbol{qhs}}$ | Proportion *awaiting diagnosis* with provider type *q* |
| $\boldsymbol{F}_{\boldsymbol{qhs}}$ | Proportion undergoing *first-line TB treatment* with provider type *q* |
| $\boldsymbol{S}_{\boldsymbol{qhs}}$ | Proportion undergoing *second-line TB treatment* with provider type *q* |
| $\boldsymbol{B}_{\boldsymbol{hs}}$ | Proportion who have temporarily dropped out of care cascade, due to missed diagnosis, pre-treatment loss-to-followup, or treatment failure |

**2. Model calibration and uncertainty**

We calibrated the model to the data summarised in table S2, performing two steps: (i) using Bayesian Markov Chain Monte Carlo (MCMC) to sample from the posterior density for the model parameters $\theta$, and (ii) using these samples to project future impact of preventive therapy, along with the uncertainty in this impact. Further details on the second step are provided below (see ‘Modelling the impact of preventive therapy’). Further details on the first step are as follows.

*Evaluating the posterior density for a given set of model parameters*

Given a set of model parameters $\theta$, we first simulated the TB epidemic to equilibrium in the absence of HIV and rifampicin-resistant TB, as well as in the absence of a public sector, assuming that in the pre-DOTS era, the standard of TB care was equivalent in both public and private sectors. We then simulated the emergence of both HIV and rifampicin resistance starting in 1990. We also simulated the expansion of high-quality public sector TB services, over a period consistent with the establishment of nationally coordinated TB programmes in each country: for example, the expansion of India’s Revised National Tuberculosis Control Programme from 1997 to 2007^21^. We then simulated the model forward in time, to the year 2018. each of the calibration targets shown in table S2 in 2018.

For a given country, we write $F_{Inc}^{\left( t \right)}(.)$ for the log-likelihood function for incidence in year $t$, and likewise $F_{Mor}^{\left( t \right)}(.)$ for the corresponding function for mortality in year $t$, both constructed by fitting log-normal probability densities to WHO estimates for annual incidence and mortality (ref). Likewise, for remaining calibration targets shown in table S2, we write $F_{Prev}^{\left( t \right)}, F_{IncHIV}^{\left( t \right)}, F_{RR}^{\left( t \right)}, F_{Notif}^{\left( t \right)}, F_{RRNotif}^{\left( t \right)}$ for the log-likelihood terms relating, respectively, to: prevalence, incidence of TB that is HIV-coinfected; the proportion of TB that is RR-TB; overall notifications; and RR-TB notifications. We used log-normal distributions for all quantities that are population rates, and beta distributions for all quantities that are proportions. Finally, we write $M_{Inc}^{\left( t \right)}(\theta)$ for the model projection for incidence in year $t$ given input parameters $\theta$, and likewise for each of the subscripts on log-likelihood terms listed above. Overall then, the full posterior density is given by:

$$\pi\left( \theta\right)=\left[ \sum_{2013}^{2018} F_{Inc}^{\left( t \right)}\left( M_{Inc}^{\left( t \right)}\left( \theta\right) \right)+F_{Mor}^{\left( t \right)}\left( M_{Mor}^{\left( t \right)}\left( \theta\right) \right) \right]+F_{Prev}^{\left( 2018 \right)}\left( M_{Prev}^{\left( 2018 \right)}\left( \theta\right) \right)+F_{IncHIV}^{\left( 2018 \right)}\left( M_{IncHIV}^{\left( 2018 \right)}\left( \theta\right) \right)+F_{RR}^{\left( 2018 \right)}\left( M_{RR}^{\left( 2018 \right)}\left( \theta\right) \right)+F_{Notif}^{\left( 2018 \right)}\left( M_{Notif}^{\left( 2018 \right)}\left( \theta\right) \right)+F_{RRNotif}^{\left( 2018 \right)}\left( M_{RRNotif}^{\left( 2018 \right)}\left( \theta\right) \right)+\sum UPr\left( \theta\right)$$

where the final term represents a sum over the log-probability densities for uniform priors on the model inputs, listed in table S3.

*Sampling from the posterior distribution*

To sample from the distribution $\pi(\theta)$, we performed adaptive MCMC^26^, a method that uses the covariance matrix of already-sampled parameters to ‘tune’ proposal distributions during the course of an MCMC chain. For each country we performed ${10}^{5}$ MCMC iterations, doubling this if required to achieve convergence. After discarding the ‘burnin’, we thinned the sample using every 100^th^ sample, to acquire roughly 1,000 samples from the posterior distribution. Using each sample we performed forward projections for the future impact of preventive therapy as described below, to yield 1,000 estimates. We estimated uncertainty by evaluating the 2.5^th^ and 97.5^th^ percentiles of these model outputs, to yield Bayesian credible intervals. Other, similar approaches in the modelling literature, for the use of Bayesian MCMC in sampling from the posterior distribution for model parameters, are available in refs.^27–29^

**3. Modelling the impact of preventive therapy**

We assumed that tests for TB infection, such as IGRA, would detect both those with latent TB infection (LTBI), denoted as $L$ in the model, and those with latent infection but imminent TB disease, denoted as $M$ in the model. In practice, those receiving preventive therapy would be drawn from both compartments, as tests such as IGRA have poor predictive value for those with LTBI who are most at risk of imminent progression to active disease^22^. We assumed that the effect of preventive therapy is to move individuals from $M$ to $L$at a certain per-capita rate, with no effect on those in $L$. That is, we assume conservatively that preventive therapy mitigates the risk of imminent TB, without necessarily curing LTBI.

For PLHIV, we modelled the per-capita rate of transition from $M$ to $L$ to reflect the number being initiated on preventive therapy. In the present analysis we instead developed a simple, alternative approach, suitable for compartmental model frameworks in general.

For household contacts, as described in the main text, modelling household structure - and its implications for recency of infection - can involve considerable increases in model dimensionality^23^. In the present analysis we instead developed a simple, alternative approach, suitable for compartmental model frameworks in general. We focused on the population implications of controlling incidence in a defined cohort. In particular, we adopted the following, three-step process:

1. *Estimating direct effects:* In a given country, we considered the cohort of household contacts of all reported TB cases. In this population, we denoted the ‘direct benefit’ $D_{HH}$ of preventive therapy as the cumulative reduction in TB incidence in the absence of any transmission effects:

$D_{HH}=NfI(1+k_{HH})c,$

where *N* is the cumulative number of TB cases notified; $f$ is the average number of household contacts per index case; $I$ is the incidence rate in the general population; $k_{HH}$ is the excess, cumulative risk of incidence associated with being a household contact; and $c$ is the effectiveness of preventive therapy in reducing incidence (where $c=1$ denotes a fully effective regimen).

Similarly, we estimated the direct benefit $D_{RG}$of preventive therapy amongst other clinical risk groups including patients receiving dialysis, organ transplantations, etc (described below), ultimately estimating the overall direct benefit $D$ as:

$D=D_{HH}+D_{RG}$

We emphasise that $D_{HH}$ and $D_{RG}$ relate only to direct effects, i.e. the benefits of preventive therapy independent of any transmission effect. Their combined direct effect therefore arises from a simple addition of the two, assuming that there is a negligible overlap between the two groups (i.e. that the number of household contacts who also belong to clinical risk groups is much smaller than the number of household contacts, as well as the number in clinical risk groups).

1. *Reproducing direct effects in the model:* We then sought to estimate the rate-of-transition from $M$ to $L$, amongst the HIV-negative population, that would yield a cumulative incidence reduction of *D* between 2020 and 2030, again in the absence of transmission effects*.* To do this we solved the governing equations with the force-of-infection $\lambda(t)$ determined, not endogenously as in the equations marked (*) above, but by fixing $\lambda\left( t \right)=\lambda^{\left( C \right)}(t),$ where $\lambda^{\left( C \right)}(t)$ is the simulated force-of-infection in the comparator scenario (i.e. with no preventive therapy). Under these conditions we varied the value of $r$ (the average rate of reversion from $M$ to $L$ due to preventive therapy), to yield a cumulative incidence reduction of *D* between 2020 and 2030, relative to the scenario $r=0$.
2. *Incorporating indirect effects:* With the value of $r$ thus determined, we simulated the ‘full’ transmission model (that is, with $\lambda(t)$ determined endogenously as in the equations marked (*) above) from 2019 to 2035, with the rate-of-transition $r$ from $M$ to $L$ in effect.

A driving parameter is the excess risk of incidence $k_{HH}$ in step (i)*,* associated with being a household contact of a TB case. To estimate this parameter we drew from a recent, longitudinal study in Vietnam^31^, as well as an ongoing, longitudinal household study in India^32^ (see table S3), both suggesting that $k_{HH}$ is approximately 7 in the region. We adopted broad uncertainty intervals around this quantity, assuming a range of 3 – 11 (table S3).

In addition to household contacts, WHO guidelines also identify those with silicosis, patients receiving dialysis or preparing for organ transplantation, as other clinical risk groups eligible for preventive therapy^11^. To model these groups we followed an approach similar to that described above for household contacts, that is aiming to estimate the population implications of the direct benefits of preventive therapy in these risk groups. For simplicity, and given a lack of data such as the population prevalence of each risk factor in each of the 11 countries in the region, we modelled the risk factors in aggregate as a single ‘risk group’, assumed to be 0.1% of the population (allowing for uncertainty ranging from 0.05% - 0.15%). By analogy to the term $D_{HH}$ described above, we estimated the direct reduction in incidence amongst clinical risk groups, $D_{RG}$, as:

$D_{RG}=MI\left( 1+k_{RG} \right)c,$

where $M$ is the overall annual number of individuals having one or more risk factors over the simulation period, $k_{RG}$ is the excess risk of TB incidence, associated with belonging to the overall risk group, and other parameters are as described above.

For the factor $k_{RG}$, we assumed a broad uncertainty interval $k_{RG}\in[2, 20]$, consistent with a 2 to 3-fold risk for silicosis^24^ and a 20-fold risk for transplantation patients^25^. See table S3 for a list of these and other parameters.

**Table S2. Epidemiological indicators for model calibration.** All rates are per 100,000 population. For simplicity the table shows only the most recent year of available data (2018), although the model was calibrated to WHO estimates for incidence and mortality from 2014 onwards ^14^. WHO no longer publishes prevalence estimates; we imputed these model inputs by calculating the most prevalence/incidence ratios available from 2014 (GTB Report 2015^14^) and applying this ratio to incidence estimates in 2018.

| Country | Prevalence rate | Incidence rate (MDR/RR-TB) | Incidence rate (HIV+TB only) | Total notification rate (+/- 10%) | MDR notification rate (+/- 10%) |
| --- | --- | --- | --- | --- | --- |
| Bangladesh | 393  [205-642] | 3.7  [2.0-5.9] | 0.45  [0.23-0.76] | 166  [149 -182] | 0.71  [0.64-0.78] |
| Bhutan | 173  [68-326] | 20  [13-28] | 0.34  [0.02-1.1] | 122  [110-134] | 8.35  [7.52-9.19] |
| DPR Korea | 174  [641-1404] | 20  [10-34] | 0.87  [0.47-1.4] | 373  [336-410] | 5.82  [5.2-6.4] |
| India | 232  [156-323] | 9.6  [5.7-15] | 6.8  [4.6-9.3] | 159  [143-175] | 3.44  [3.1-3.8] |
| Indonesia | 512  [406-631] | 8.8  [6.2-12] | 7.9  [3.3-14] | 213  [192-234] | 1.56  [1.41-1.72] |
| Maldives | 45  [20-79] | 0.73  [0.05-2.3] | 0.24  [0-1.2] | 27  [24-30] | 0.39  [0.35-0.43] |
| Myanmar | 436  [361-511] | 21  [14-30] | 29  [19-41] | 260  [234-286] | 4.93  [4.4-5.4] |
| Nepal | 205  [97-353] | 5.0  [2.8-8.0] | 1.4  [1.1-1.6] | 116  [104-127] | 1.42  [1.28-1.56] |
| Sri Lanka | 97  [50-161] | 0.12  [0.01-0.37] | 0.27  [0.16-0.4] | 42  [38-46] | 0.06  [0.05-0.07] |
| Thailand | 211  [144-292] | 5.7  [3.3-8.8] | 15  [12-20] | 125  [113-138] | 1.31  [1.18-1.44] |
| Timor Leste | 820  [426-1340] | 19  [6.4-38] | 6.1  [3.5-9.5] | 308  [277-339] | 0.95  [0.85-1.04] |
| SEAR | 257[137-440] | 9.2[6.3-13] | 7.1[5.4-9.0] | 170[153-187] | 2.90[2.61-3.19] |

**Table S3. List of regional-level parameters used in the model.** See table S4 for additional, country-level parameters. Parameters whose estimates can be found in table S4 are indicated as such. Where parameters are subject to uncertainty, the assumed intervals are given in parentheses. All remaining parameters are held fixed, and are not expected to play an important role in model estimates. Footnotes: (a) Unless other country-specific information was available, we drew from a recent systematic review in India, of the public care cascade^1^. An exception is Bangladesh, where treatment initiation rates are estimated as 99%. (b) Given a lack of systematic evidence quantifying the care cascade in the private sector in SEAR, we assumed the parameters specified here for each country, with the exception of Thailand, where the private healthcare sector has a good quality of TB care, but tends not to notify TB. Here we assumed the same parameters for the care cascade as in the public sector. (c) IPT and 3HP have similar effectiveness in reducing TB, amongst those completing the regimens. However, the available evidence shows that 3HP, being a shorter, simpler and safer regimen than IPT, has substantially higher completion rates. We thus adjusted downwards the assumed value for effectiveness of IPT, to reflect the lower rates of completion that would be expected in practice.

| Parameter name | | | Symbol | Value | Note/Source | |
| --- | --- | --- | --- | --- | --- | --- |
| *Natural history parameters* | | | | | | |
| Average infections per infectious TB case per year | Drug-susceptible TB | | $\beta$ | Calibrated to yield incidence and prevalence for given country setting (see table S4) | | |
|  | Drug-resistant TB | | $\beta_{MDR}$ |  |  |  |
| Proportion of infections undergoing rapid progression $\boldsymbol{(}\boldsymbol{f}_{\boldsymbol{h}}\boldsymbol{)}$ | HIV -ve | | $f_{0}$ | 0.1 (0.05 -0.15) | Ref.^48^ | |
|  | HIV +ve | | $f_{1}$ | ${2.5* f}_{0}$ | Ref.^49^ | |
| Rate of breakdown to imminent disease $\boldsymbol{(}\boldsymbol{g}_{\boldsymbol{h}}\boldsymbol{)}$ | HIV -ve | | $g_{0}$ | 0.0027 y^-1^(0.0005-0.005) | Ref.^39^ | |
|  | HIV +ve | | $g_{1}$ | ${23*g}_{0}$ | Ref.^39^ | |
| Per-capita hazard of progression from ‘imminent TB’ compartment to active disease | | | *h* | 0.5 | Follows from definition of ‘imminent’ disease as those at risk of developing disease within 2 years | |
| Per-capita relapse rate | | | $\rho$ | 0.0017 y-1 | Corresponding to 10% lifetime risk^40,41^ | |
| Per-capita rate of self-cure, active TB | | | $\sigma$ | 0.166 y-1 | Together corresponds to 50% spontaneous cure, 50% mortality in average of 3 years 15 | |
| Per-capita mortality hazard rate, active TB | | | $\mu_{TB}$ | 0.166 y-1 |  |  |
| *Care cascade parameters, first-line* | | | | | | |
| Per-capita rate of first presentation to a provider following onset of symptoms | | | *c* | Governs the initial patient delay: calibrated together with $\beta, \beta_{MDR}$to yield incidence and prevalence (see table S4) | | |
| Probability that a TB patient visits a provider of type *q,* per careseeking attempt | | | $p_{q}$ | Calibrated for simulated treatment initiations to match reported notifications (see table S4) | | |
| Per-capita rate of offering a diagnosis | | | *d* | 52 y^-1^ | Assumption: corresponds to an average of 1 week to arrive at a diagnosis | |
| Probability of successful diagnosis and treatment initiation with provider type *q* | | | $u_{q}$ | Calculated using $u_{q}=p_{q}^{\left( Dx \right)}p_{q}^{\left( Tx \right)},$ for values of $p_{q}^{\left( Dx \right)} , p_{q}^{\left( Rx \right)}$ given below | | |
| Per-capita rate of default from treatment from provider type *q* | | | $\delta_{q}$ | Calculated using $\delta_{q}=\tau^{\left( FL \right)}p_{q}^{\left( FL \right)}/(1-p_{q}^{\left( FL \right)}),$for values of $\tau^{\left( FL \right)}, p_{q}^{\left( Dx \right)}$given below | | |
| Probability of correct TB diagnosis per visit to a provider | | NTP provider | $p_{0}^{(Dx)}$ | 0.83 (0.8 – 0.85) | Ref.^1^ | |
|  |  | Non-NTP provider | $p_{1}^{(Dx)}$ | 0.7 (0.6 – 0.8) | Assumed | |
| Proportion of diagnosed cases initiating treatment | | NTP provider | $p_{0}^{(Rx)}$ | 0.88 (0.85 – 0.9) | Ref.^1^ | |
|  |  | Non-NTP provider | $p_{1}^{(Rx)}$ | 0.7 (0.6 – 0.8) | Assumed | |
| Proportion completing first-line treatment | | NTP provider | $p_{0}^{(FL)}$ | Drawn from WHO country reports^14^ | | |
|  |  | Non-NTP provider | $p_{1}^{(FL)}$ | 0.6 (0.5 – 0.7) | Assumed | |
| *Care cascade, second-line* | | | | | | |
| Probability of provider offering second-line testing at point of TB diagnosis (in absence of Xpert) | | NTP provider | $v_{0}$ | 0.2 | From baseline data of GeneXpert demonstration study in India^42^ | |
|  |  | Non-NTP provider | $v_{1}$ | 0.1 | Assumption | |
| Proportion of first-line treatment failures being switched to second-line treatment | | NTP provider | $w_{0}$ | Calibrated for simulated, second-line treatment initiations to match reported RR/MDR notifications 16 (see table S4) | | |
|  |  | Non-NTP provider | $w_{1}$ | 0.1 | Assumption | |
| Proportion treatment success, second-line treatment | | NTP provider | $p_{0}^{(SL)}$ | 0.5 | Taken from country reports where available^14^ | |
|  |  | Non-NTP provider | $p_{1}^{(SL)}$ | 0.2 | Assumption | |
| *Other care parameters* | | | | | | |
| Duration of first-line regimen | | | $\tau^{\left( FL \right)}$ | 2 y-1 | Corresponds to a 6 month regimen^43^ | |
| Duration of second-line regimen | | | $\tau^{\left( SL \right)}$ | 0.5 y^-1^ | Corresponds to a 2 year regimen^43^ | |
| Rate of repeat care seeking for patients who have dropped out of care cascade | | | $\gamma$ | 6 – 24 y-1 | Yields an interval between careseeking episodes with uncertainty range of 2 weeks to 2 months^44^. | |
| *Population structure* | | | | | | |
| Per-capita birth rate | | | *b* | Selected to yield projected population growth | | |
| Per-capita ‘background’ mortality hazard | | | $\mu$ | 1/66 | Corresponding to a TB-free life expectancy of 66 years for India (World Bank, adjusted to country-specific data) | |
| Reduction of force of infection owing to previous infection | | | $c_{i}$ | 0.85 | | Ref.^45^ |
| Relative transmission efficiency for HIV +ve patients | | | $\kappa$ | 0.7 | | Assumption |
| *Preventive therapy parameters* | | | | | | |
| Incidence rate ratio, household contacts vs general population | | | $k_{HH}$ | 7.0 (3.0 – 11.0) | | Assumption based on Ref.^31,32^ |
| Incidence rate ratio, other clinical risk groups vs general population | | | $k_{RG}$ | 11.0 (2.0 – 20.0) | | Assumption based on Ref.^24,25^ |
| Effectiveness of preventive therapy | | | $c$ | 0.3 (for IPT)  0.6 (for 3HP) | | Ref.^15,17,46^ |
| Average number of household contacts per index case | | | $f$ | Varies by country, from 3.7 (Thailand) to 5.8 (Timor-Leste) | | Ref.^47^ |
| Number belonging to clinical risk groups other than household contacts | | | *M* | 0.1% (0.05% - 0.15% of country population | | Assumption |

**Table S4: Summary of country-wise parameter estimates**. Numbers in brackets show the uncertainty ranges used in the simulations (for input parameters), or Bayesian credible intervals (for parameters being estimated). Parameters are as follows: *β* is the average number of infections per drug-susceptible (DS) TB case per year; *β_mdr_* is the average number of infections per MDR-TB case per year; *r* is the rate of initial careseeking; *p_NTP_* is the proportion of TB cases visiting an NTP provider at each care seeking attempt (rather than the private sector); $p_{DST}$ is the proportion of TB diagnoses receiving a drug susceptibility test; *p_hiv_* is the proportion of incident TB cases that are HIV infected; *μ_non-NTP_* is the mortality hazard of TB patients being managed by non-NTP providers (in units of inverse years); *d_β_* is the annual decline rate of *β*; *and d_mor_* is the annual decline rate of TB mortality, both of the latter two having units of inverse years.

| Country | *β* | *β_mdr_* | *r* | *p_NTP_* | $\boldsymbol{p}_{\boldsymbol{DST}}$ | *p_hiv_* | *μ_non-NTP_* | *d_β_* | *d_mor_* |
| --- | --- | --- | --- | --- | --- | --- | --- | --- | --- |
| Bangladesh | 22.7  [7.6-37.8] | 6.6[2.6-9.5] | 1.0[0.5-1.8] | 0.85[0.58-0.99] | 0.03[0.02- 0.06] | 0.002[0.001-0.003] | 0.22[0.11-0.44] | 0.89[0.8-0.99] | 0.98[0.93-1.03] |
| Bhutan | 27.3[12.2-38.3] | 8.6[5.9-9.9] | 6.4[2.1-9.8] | 0.45[0.25- 0.77] | 0.11 [0.07-0.19] | 0.002[0.0005-0.005] | 0.15[0.07-0.27] | 0.88 [0.8-1.0][ | 0.99[ 0.92 -1.1] |
| DPR Korea | 37.1 [27.7 -49.6] | 2.1 [1.2 -5.4] | 3.07 [0.86- 4.78] | 0.49 [0.35- 0.74] | 0.11 [0.08 – 0.17] | 0.0002 [0.0001 -0.0004] | 0.14 [0.06 -0.26] | 1.29 [1.09 -1.4] | 1.0 [0.97-1.04] |
| India | 22.6 [12.7 -37.2] | 7.8 [4.6- 9.9] | 2.4 [1.5- 4.3] | 0.48 [0.36 -0.70] | 0.08 [0.05- 0.11] | 0.004 [0.002 -0.006] | 0.24 [0.1 – 0.35] | 0.96 [0.90 – 1.0] | 0.99[ 0.97 – 1.03] |
| Indonesia | 15.6 [11.5 – 22.7] | 4.3 [2.5-5.3 | 0.84[ 0.62 -1.34] | 0.47 [0.35-0.60] | 0.03[0.025 -0.041] | 0.003 [0.002-0.005] | 0.12 [0.09- 0.21] | 0.99[ 0.96 – 1.01] | 0.98 [0.95- 0.99] |
| Maldives | 7.9[7.4 -8.4] | 0.77[ 0.69- 1.35] | 4.95 [4.92 – 4.99] | 0.37 [0.35 – 0.40] | 0.07 [0.05- 0.08] | 0.00004 [0.00003- 0.00004] | 0.06 [0.05 -0.07] | 1.033 [1.03-1.07] | 0.82 [0.81 -0.823] |
| Myanmar | 21.6[ 9.1- 39.3] | 3.7 [2.5 – 5.9] | 2.4 [0.95-4.2] | 0.4 [0.3-0.7] | 0.05 [0.04 – 0.08] | 0.01 [0.006 – 0.015] | 0.11 [0.05 -0.29] | 0.98 [0.95 -1.03] | 1.0 [0.95 -1.05] |
| Nepal | 28.2 [15.7 -46.6] | 5.2 [3.5 – 7.3] | 4.0[1.6 – 7.1] | 0.55 [0.42 – 0.69] | 0.06 [0.04 – 0.09] | 0.0008 [0.0007 -0.001] | 0.13 [0.07 – 0.24] | 0.99 [0.94 – 1.03] | 1.03 [0.97 – 1.1] |
| Sri Lanka | 15.5[ 12.3 – 18.6] | 0.08 [0.005 – 0.25] | 3.9 [2.8 – 4.9] | 0.35 [0.28 – 0.46] | 0.006 [0.005 -0.009] | 0.0003 [0.0002- 0.0005] | 0.08 [0.06-0.10] | 0.95 [0.88 -1.01] | 0.98 [0.92 – 1.04] |
| Thailand | 6.8 [4.4 – 12.3] | 3.3 [2.1 – 4.7] | 0.99 [0.62 – 1.61] | 0.68 [0.44 – 0.94] | 0.04 [0.03 – 0.05] | 0.011 [0.007 – 0.015] | 0.13 [0.06 – 0.19] | 1.0 [0.96 -1.04] | 0.96 [0.94 – 0.99] |
| Timor Leste | 4.0 [2.1 – 8.8] | 3.3 [2.1 – 4.8] | 3.7 [1.2 – 8.2] | 0.04[0.02 – 0.08] | 0.04 [0.02 – 0.06] | 0.002 [0.001 -0.003] | 0.16 [0.08 -0.32] | 1.06 [1.01 -1.12] | 0.99 [0.93 -1.04] |

**Table S5. Impact on cumulative TB incidence** by country, and for the whole region, from 2019-2030. While results in the main text show impact in terms of reductions in annual incidence rates (in line with the End TB goals), this table summarises impact attributable to preventive therapy alone, in terms of reductions in *cumulative* TB cases between 2019 and 2030. In this table, estimates under ‘status quo comparator’ relate to the difference between blue and orange curves in the left-hand panel of Figure 2; estimates under ‘improved TB cascade comparator’ relate to the difference between green and yellow curves in the right-hand panel of Figure 2.

| Cases averted (2019-2030) | Status quo comparator | | Improved TB cascade comparator | |
| --- | --- | --- | --- | --- |
|  | **Number of TB cases averted**  **(thousands)** | **Pct reduction in cumulative incidence** | **Number of TB cases averted (thousands)** | **Pct reduction in cumulative incidence** |
| Bangladesh | 138[129-194] | 3.8[3.2-4.5] | 136[122-158] | 3.7[3.1-5.0] |
| Bhutan | 0.33[0.27-0.48] | 2.8[2.3-3.2] | 0.32[0.28-0.39] | 2.8[2.4-3.3] |
| DPR Korea | 144[84-196] | 4.5[2.5-9.6] | 101[42-146] | 5.5[2.0-10.6] |
| India | 1450[1200-1925] | 5.1[4.0-6.4] | 1394[1160-1661] | 5.2[4.2-6.4] |
| Indonesia | 555[451-735] | 5.7[4.8-6.6] | 521[451-625] | 6.3[5.4-7.1] |
| Maldives | 0.03[0.02-0.1] | 1.6[1.4-2.4] | 0.02[0.01-0.03] | 1.4[0.9-1.7] |
| Myanmar | 167[138-323] | 8.2[6.3-11.1] | 202[155-353] | 10.4[7.5-12.7] |
| Nepal | 18.2[15.0-30.4] | 3.6[3.3-4.1] | 17[14-22] | 3.8[3.2-4.6] |
| Sri Lanka | 2.2[1.9-2.9] | 1.6[1.3-2.0] | 2.5[2.0-3.1] | 2.1[1.5-2.3] |
| Thailand | 92[64-171] | 7.4[5.7-9.0] | 70[58-94] | 7.0[5.8-8.5] |
| Timor Leste | 14.0[10.6-20.4] | 10.6[6.8-14.1] | 8.5[6.8-11.4] | 11.8[9.7-14.3] |
| SEAR | 2642[2274-3194] | 5.2[4.6-6.1] | 2472[2207-2879] | 5.6[4.8-6.5] |

**Table S6. Impact on cumulative TB mortality** by country, and for the whole region, from 2019-2030. Similar to table S5, this table summarises reductions in *cumulative* TB mortality between 2019 and 2030, attributable to preventive therapy alone.

| Deaths averted (2019-2030) | Status quo comparator | | Improved TB cascade comparator | |
| --- | --- | --- | --- | --- |
|  | **Number of TB deaths averted (thousands)** | **Pct reduction in cumulative TB mortality** | **Number of TB deaths averted (thousands)** | **Pct reduction in cumulative TB mortality** |
| Bangladesh | 15.5[7.4-28.9] | 3.1[2.4-4.1] | 8.5[3.5-17.9] | 2.7[1.9-3.6] |
| Bhutan | 0.03[0.01-0.07] | 2.3[1.8-3.03] | 0.02[0.01-0.04] | 2.1[1.4-2.9] |
| DPR Korea | 13.8[7.2-25.8] | 3.9[2.4-8.7] | 6.4[1.9-15.0] | 4.4[1.6-8.6] |
| India | 206[146-278] | 4.4[3.5-5.6] | 123[88.7-187.3] | 3.9[3.2-4.9] |
| Indonesia | 40[33-50] | 4.4[3.7-5.1] | 24.4[19.2-33.5] | 4.2[3.6-4.8] |
| Maldives | 0.0001[0.00006-0.00014] | 0.7[0.6-1.1] | 0.00005[0.00003- 0.00006] | 0.6[0.4-0.7] |
| Myanmar | 15.3[9.7-26.3] | 6.6[5.2-8.8] | 11.4[5.8-16.9] | 7.4[5.1-9.5] |
| Nepal | 2.6[1.2-4.8] | 3.5[2.8-4.1] | 1.6[0.5-3.4] | 3.2[2.4-4.1] |
| Sri Lanka | 0.09[0.05-0.19] | 1.3[1.0-1.7] | 0.08[0.03-0.15] | 1.6[0.9-1.9] |
| Thailand | 4.2[2.5-8.1] | 5.5[4.3-7.2] | 2.2[1.2-4.5] | 4.5[3.7-6.0] |
| Timor Leste | 1.95[0.87-4.5] | 8.6[5.9-11.6] | 0.65[0.24-1.86] | 7.9[5.5-10.6] |
| SEAR | 303[237-369] | 4.4[3.8-5.2] | 179[139-243] | 4.0[3.4-4.9] |

**Table S7. Total impact of improved TB cascade and preventive therapy.** Results presented elsewhere in this study refer to the impact attributable solely to preventive therapy. In the ‘improved TB cascade’ comparator, these results are thus incremental to the impact of improving the TB cascade. Presented here are results for the *combined* impact of both cascade improvements and preventive therapy.

| Improved TB cascade + preventive therapy | Reductions in annual rates  (to complement Table 1 in main text) | | Reductions in cumulative burden between 2019 – 2030 (to complement Tables S5, S6) | |
| --- | --- | --- | --- | --- |
|  | **% Reduction in annual incidence rate**  **(2030 relative to 2015)** | **% Reduction in annual TB deaths**  **(2030 relative to 2015)** | **Number of TB cases averted (thousands)** | **Number of TB deaths averted (thousands)** |
| Bangladesh | 46.3[39.0-57.2] | 74.5[41.3-90.8] | 342[224-991] | 154[100-235] |
| Bhutan | 46.5[24.6-54.9] | 67.1[-6.1-91.0] | 0.70[0.41-3.12] | 0.35[0.20-0.67] |
| DPR Korea | -6.7[-63.1-33.3] | 35.6[-54.2-71.2] | 1026[313-3999] | 140[62-479] |
| India | 44.4[33.9-50.7] | 60.3[41.7-70.3] | 3479[2182-6297] | 1642[1388-1986] |
| Indonesia | 47.4[33.7-57.8] | 74.6[61.8-84.3] | 2254[1170-3745] | 370[289-452] |
| Maldives | 17.8[-56.5-35.9] | 97.1[96.1-97.7] | 0.41[0.17-1.79] | 0.002[0.001-0.004] |
| Myanmar | 48.4[8.2-61.8] | 70.3[53.5-86.6] | 416[231-1426] | 88[54-226] |
| Nepal | 42.2[8.4-53.5] | 30.6[-95.8-83.7] | 92[44-235] | 24[13-48] |
| Sri Lanka | 40.5[27.3-50.1] | 70.4[31.8-85.5] | 13.3[6.7-31.6] | 1.3[0.8-2.3] |
| Thailand | 42.7[4.2-56.6] | 79.0[41.3-91.0] | 330[168-898] | 30.6[19.6-60.5] |
| Timor Leste | 41.5[-48.5-72.3] | 64.9[-8.1-91.2] | 71.6[38.4-148.3] | 14.7[7.4-36.6] |
| SEAR | 43.3[37.4-47.8] | 63.2[52.7-71.5] | 8911[6310-12337] | 2535[2217-2996] |

**Figure S1: Calibration results to incidence and mortality**. Dots and error bars show WHO estimates of incidence and mortality; solid blue lines show model-simulated results; and shaded regions show uncertainty in model projections (95% credible intervals).


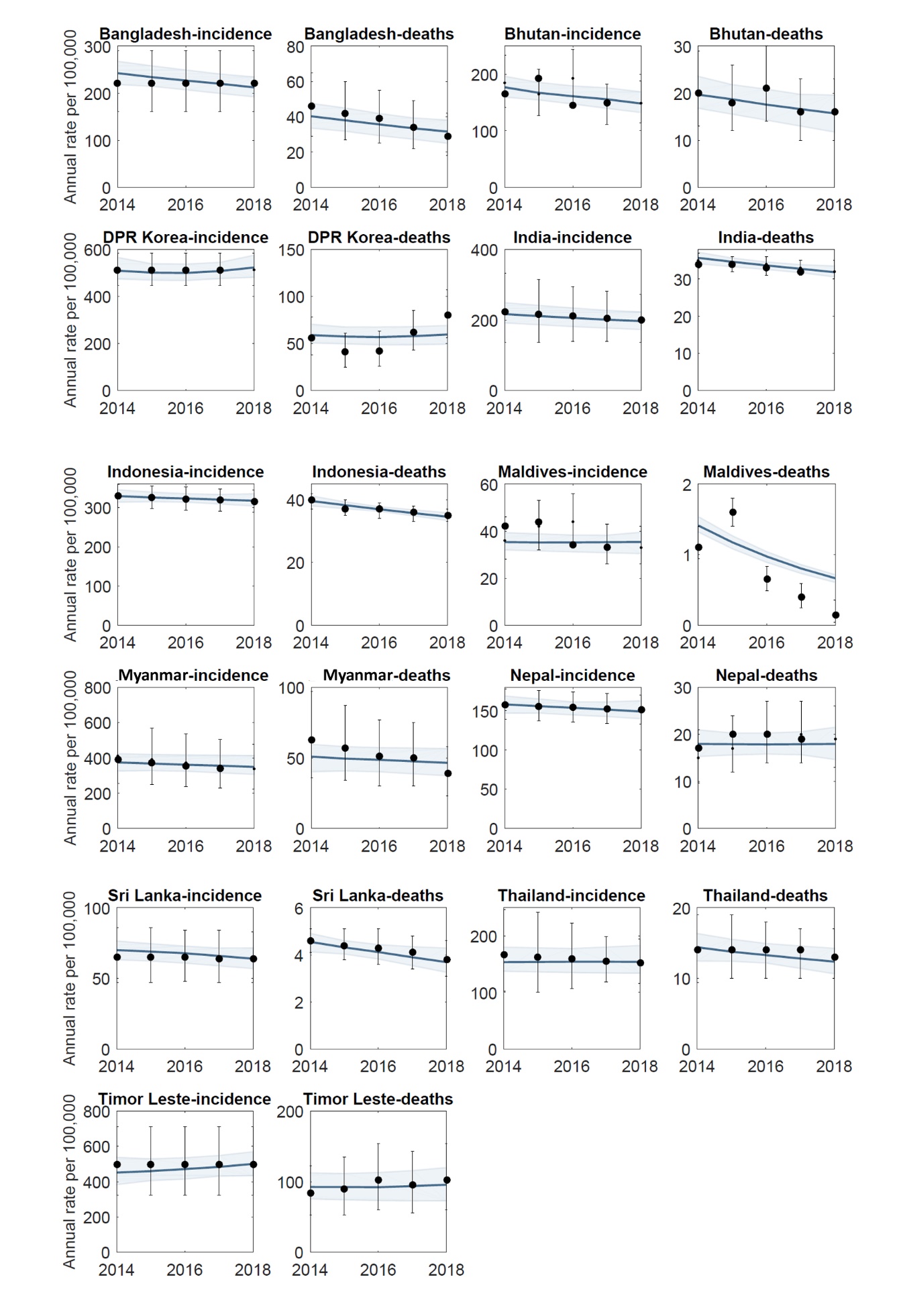


**Figure S2: Calibration results to additional indicators.** Black indicates estimates and data to which the model is calibrated, while red indicates model simulation outputs.


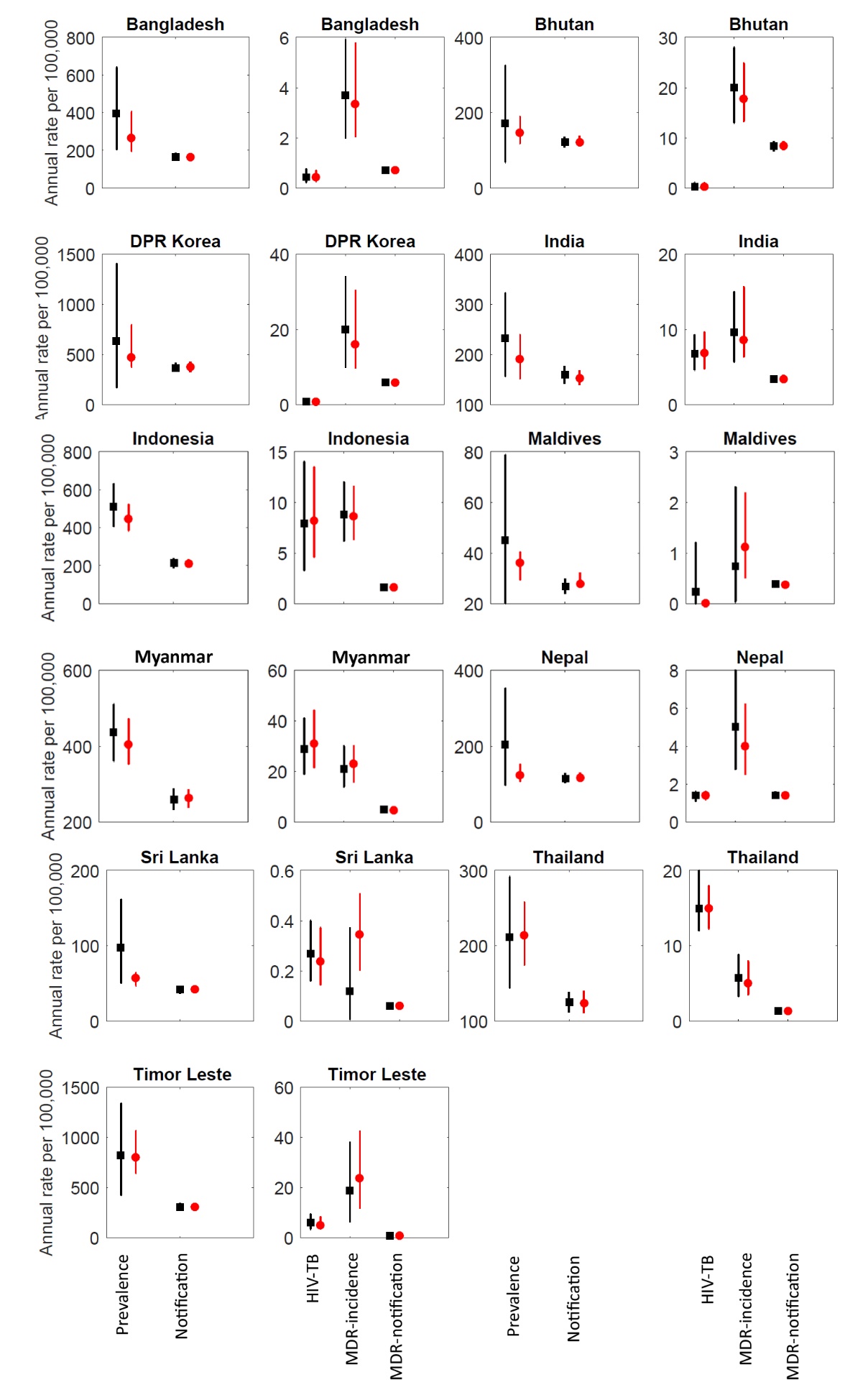


**Figure S3:** Sensitivity analysis to assumptions for coverage and effectiveness of preventive therapy. In the main text we present results under the assumption that (after a three-year scale-up), all household contacts and clinical risk groups receive preventive therapy, with efficacy matching that observed in clinical trials. Here we simulate a range of less idealised scenarios, defining ‘effective coverage’ as a product of the actual coverage of preventive therapy (proportion of eligible population receiving it), and its efficacy relative to clinical trials. The left-hand panel shows incidence impact for a range of effective coverage, while the right-hand panel shows mortality impact, both panels illustrating a roughly proportional relationship between effective coverage and impact.


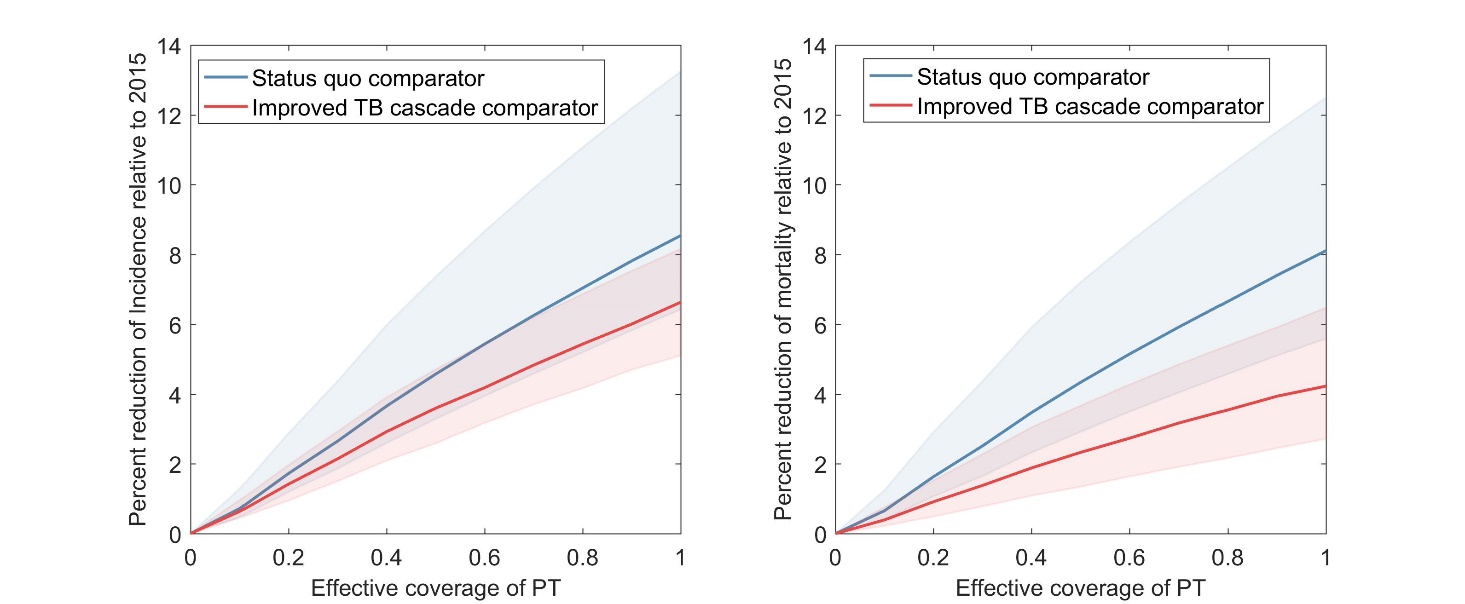


**Figure S4. Sensitivity analysis to model parameters**. The model shows partial rank correlation coefficients of each of the model inputs (parameters and data) against a selected model outcome, the percent reduction in incidence rates in 2030 relative to 2015. Inputs are listed in order of decreasing sensitivity from top to bottom.


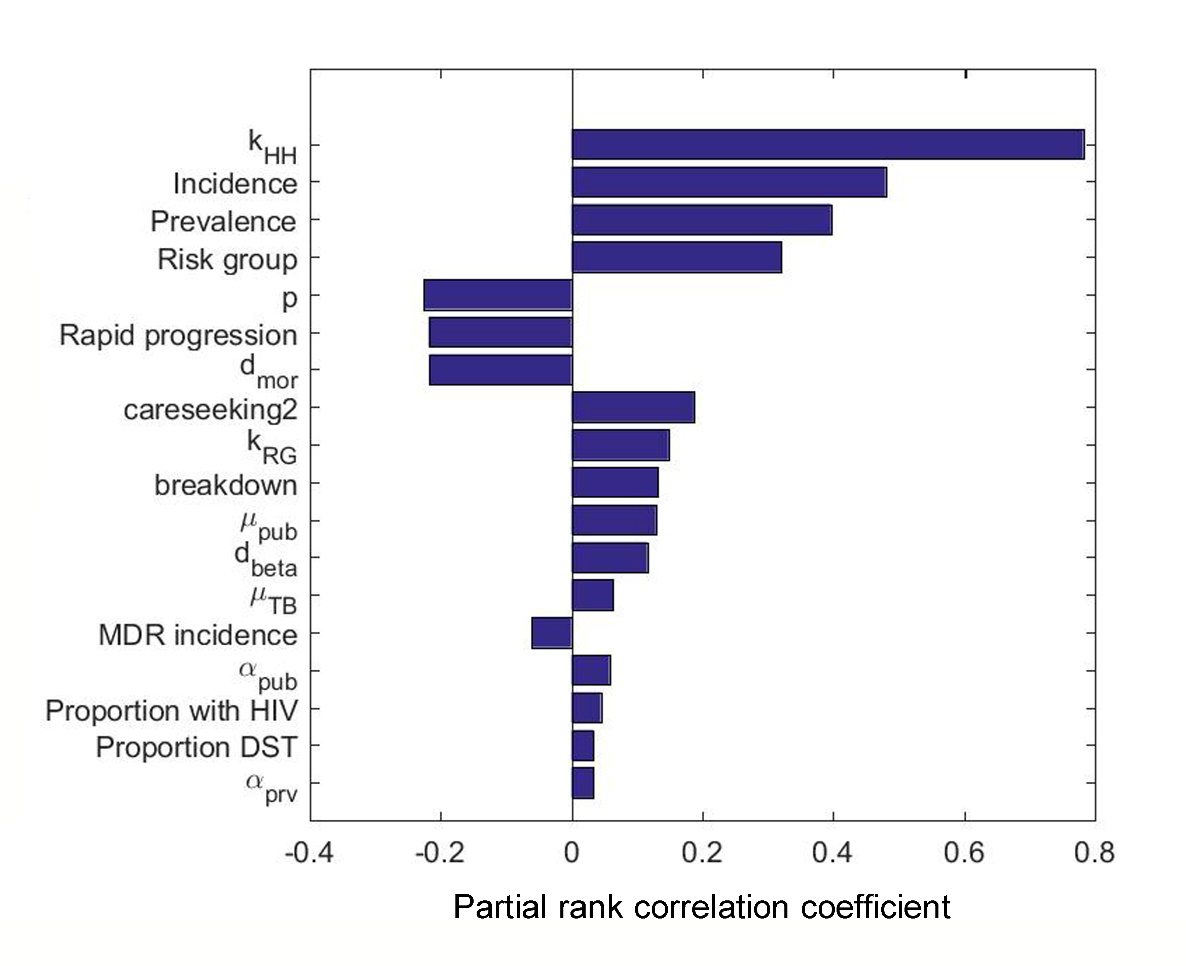

Supplement: Supplementary file 1 — Additional file 1 : Figure S1. Calibration results to incidence and mortality. Figure S2. Calibration results to additional indicators. Figure S3. Sensitivity analysis to assumptions for coverage and effectiveness of preventive therapy. Figure S4. Sensitivity analysis to model parameters. Table S1. List of state variables used in the model. Table S2. Epidemiological indicators for model calibration. Table S3. List of regional-level parameters used in the model. Table S4. Summary of country-wise parameter estimates. Table S5. Impact on cumulative TB incidence by country, and for the whole region, from 2019-2030. Table S6. Impact on cumulative TB mortality by country, and for the whole region, from 2019-2030. Table S7. Total impact of improved TB cascade and preventive therapy. [file 12916_2020_1651_MOESM1_ESM.docx]
